# Diagnostic efficacy of serum presepsin for postoperative infectious complications: a meta-analysis

**DOI:** 10.3389/fimmu.2023.1320683

**Published:** 2023-12-12

**Authors:** Chun-Ying Lu, Chia-Li Kao, Kuo-Chuan Hung, Jheng-Yan Wu, Hui-Chen Hsu, Chia-Hung Yu, Wei-Ting Chang, Ping-Hsun Feng, I-Wen Chen

**Affiliations:** ^1^ Department of Anesthesiology, Chi Mei Medical Center, Tainan, Taiwan; ^2^ Department of Anesthesiology, E-Da Hospital, I-Shou University, Kaohsiung, Taiwan; ^3^ Department of Nutrition, Chi Mei Medical Center, Tainan, Taiwan; ^4^ Department of Otolaryngology, Kuang Tien General Hospital, Taichung, Taiwan; ^5^ School of Medicine and Doctoral Program of Clinical and Experimental Medicine, College of Medicine and Center of Excellence for Metabolic Associated Fatty Liver Disease, National Sun Yat-sen University, Kaohsiung, Taiwan; ^6^ Division of Cardiology, Department of Internal Medicine, Chi Mei Medical Center, Tainan, Taiwan; ^7^ Department of Biotechnology, Southern Taiwan University of Science and Technology, Tainan, Taiwan; ^8^ Department of Anesthesiology, Chi Mei Medical Center, Liouying, Tainan City, Taiwan

**Keywords:** presepsin, procalcitonin, C-reactive protein, postoperative, infectious complications, meta-analysis

## Abstract

**Background:**

Postoperative infectious complications (PICs) are major concerns. Early and accurate diagnosis is critical for timely treatment and improved outcomes. Presepsin is an emerging biomarker for bacterial infections. However, its diagnostic efficacy for PICs across surgical specialties remains unclear.

**Methods:**

In this study, a systematic search on MEDLINE, Embase, Google Scholar, and Cochrane Library was performed on September 30, 2023, to identify studies that evaluated presepsin for diagnosing PICs. PIC is defined as the development of surgical site infection or remote infection. Pooled sensitivity, specificity, and hierarchical summary receiver operating characteristic (HSROC) curves were calculated. The primary outcome was the assessment of the efficacy of presepsin for PIC diagnosis, and the secondary outcome was the investigation of the reliability of procalcitonin or C-reactive protein (CRP) in the diagnosis of PICs.

**Results:**

This meta-analysis included eight studies (n = 984) and revealed that the pooled sensitivity and specificity of presepsin for PIC diagnosis were 76% (95% confidence interval [CI] 68%–82%) and 83% (95% CI 75%–89%), respectively. The HSROC curve yielded an area under the curve (AUC) of 0.77 (95% CI 0.73–0.81). Analysis of six studies on procalcitonin showed a combined sensitivity of 78% and specificity of 77%, with an AUC of 0.83 derived from the HSROC. Meanwhile, data from five studies on CRP indicated pooled sensitivity of 84% and specificity of 79%, with the HSROC curve yielding an AUC of 0.89.

**Conclusion:**

Presepsin exhibits moderate diagnostic accuracy for PIC across surgical disciplines. Based on the HSROC-derived AUC, CRP has the highest diagnostic efficacy for PICs, followed by procalcitonin and presepsin. Nonetheless, presepsin demonstrated greater specificity than the other biomarkers. Further study is warranted to validate the utility of and optimize the cutoff values for presepsin.

**Systematic review registration:**

https://www.crd.york.ac.uk/prospero/, identifier CRD42023468358.

## 1 Introduction

Postoperative infectious complications (PICs), encompassing surgical site and remote infections (e.g., pneumonia), are major concerns after surgical procedures and occur in up to 7.2%–29.5% of patients (1–3). These complications result in increased patient morbidity and mortality, prolonged hospital stays, and increased healthcare costs (3–8). Furthermore, a retrospective study involving 186 patients who underwent colorectal surgery described postoperative infection as an independent predictor of diminished 5-year survival (1). Therefore, early and accurate diagnosis of postsurgical infections is critical for timely clinical management and improved outcomes. While PICs are generally identified through a clinical diagnosis, certain complications, such as anastomotic leaks, may manifest late, after hospital discharge (9). Consequently, the predictive value of clinical symptoms during the postoperative period is insufficient for detecting all infectious complications. The use of prophylactic antibiotics postsurgery might be beneficial in reducing complications from postoperative infections. However, inappropriate or excessive use of antibiotics can lead to the evolution of antibiotic resistance and infection due to *Clostridium difficile* (10). Thus, it is advocated that antibiotics be used only when necessary and suitable, and initiatives should be taken to endorse prudent antibiotic usage (11). The identification of ideal biomarkers for the early detection of PICs is important, enabling the prompt commencement of suitable antibiotics.

Presepsin (soluble CD14 subtype) is a 13-kDa truncated fragment derived from the CD14 membrane protein and is released into circulation during monocyte activation upon recognition of lipopolysaccharides from bacterial cell walls (12). Several studies have recently reported that presepsin may be used to predict sepsis or PICs (1, 13–16). Compared with other conventional biomarkers, presepsin has distinct advantages, including an early rise of infection onset within 2 h and rapid normalization after recovery (17). Furthermore, it is minimally affected by noninfectious inflammatory conditions. These desirable characteristics potentiate its clinical utility for the early diagnosis of bacterial infections. While individual studies have evaluated presepsin for PICs, its diagnostic accuracy may widely vary depending on surgical procedure, measurement timing, and threshold values (1, 15, 18, 19). A comprehensive synthesis of existing data regarding the diagnostic precision and clinical value of presepsin for PICs across surgical specialties is lacking. Therefore, we conducted a meta-analysis of all available studies to establish the overall diagnostic efficacy and reliability of presepsin for the early diagnosis of PICs after diverse surgical procedures. In addition, we compared its accuracy with those of other conventional biomarkers, such as C-reactive protein (CRP) and procalcitonin.

## 2 Methods

### 2.1 Protocol registration

This meta-analysis strictly adhered to the guidelines delineated by the Preferred Reporting Items for Systematic Reviews and Meta-analyses. The protocol for this meta-analysis was officially registered on September, 2023, in the PROSPERO database (registration number: CRD42023468358), ensuring transparency and adherence to predefined methodological specifications.

### 2.2 Search strategy

Two research members independently performed exhaustive searches across several scientific databases, namely, MEDLINE, Embase, Google Scholar, and Cochrane Library. The search spectrum encompassed all relevant studies available from database inception until September 30, 2023; no restrictions on language or publication date were imposed, thereby ensuring a comprehensive inclusion of available literature. The search methodology involved the use of both text words and medical subject headings, focusing on the following key terminologies: “presepsin,” “sCD14-ST,” “soluble CD14 subtype,” “surgical,” “surgery,”, “infection,” “sensitivity,” and “specificity.” The exhaustive search strategy for one of the databases (i.e., MEDLINE) is presented in detail in **Supplementary Table 1**, allowing for transparency and replicability of the search process. Similar strategies were adapted for other databases. Furthermore, the reference lists of the identified studies were thoroughly examined to ensure the inclusion of additional studies that were potentially overlooked during the initial search.

### 2.3 Inclusion criteria

Only observational studies (prospective or retrospective cohort, case–control) were included in current meta-analysis. Two independent reviewers screened the titles and abstracts of the retrieved studies for potential eligibility. Then, full texts were assessed to confirm final study inclusion based on Population, Intervention, Comparison, Outcomes, Time (PICOT) framework:

1. P (Population): The population of interest is adult individuals subjected to any form of surgery, including elective or emergent.
2. I (Intervention): The index test is measurement of serum presepsin levels after surgery.
3. C (Comparison): The standard of care was used as the reference standard.
4. O (Outcome)- The outcome is diagnostic efficacy of serum presepsin for PICs (e.g., surgical site infection [SSI], pneumonia, and urinary tract infection).
5. T (Time) - The diagnostic efficacy of presepsin in identifying PICs is assessed during the first seven days following surgery.

Studies were excluded if (1) they are reviews, case reports, conference abstracts, nonhuman studies, non-peer-reviewed article; (2) presepsin was not collected from serum (e.g., from synovial fluid); (3) they focused on pediatric population or patients with established infection before surgery; (4) they included patients with end-stage renal disease; and (5) there is insufficient data (e.g., sensitivity, specificity, number of events) to calculate true positive (TP), false positive (FP), false negative (FN), and true negative (TN). Any disagreements were resolved through consensus or consultation with a third reviewer if needed.

### 2.4 Data extraction

Two reviewers independently extracted relevant data using a predefined data extraction form. The extracted information included (1) study characteristics (author, year, design, country, sample size), (2) patient characteristics (age, gender), (3) surgery details (type of surgery), (4) biomarker details (cutoff values of biomarkers [i.e., presepsin, CRP, and procalcitonin) for infection complications and timing of measurement (e.g., postoperative day 3), and (5) sensitivity and specificity values. Authors of the included studies were contacted for any missing or unclear information.

### 2.5 Definition and outcomes

PIC was defined as the development of SSIs or remote infections (e.g., pneumonia and urinary tract infection) (20). The primary outcome was the assessment of the diagnostic efficacy of presepsin for PIC, and the secondary outcome was the investigation of the diagnostic efficacy of CRP or procalcitonin in the diagnosis of PICs. The criteria and cutoff values for biomarkers used to diagnose PICs were established based on individual studies, rather than depending on a single criterion.

### 2.6 Quality assessment

QUADAS-2 was used to assess risk of bias and applicability concerns in four domains: patient selection, index test, reference standard, and flow and timing. Each domain was judged as low, high, or unclear risk of bias/concerns. Two reviewers independently assessed the study quality.

### 2.7 Data synthesis and analysis

Data were organized on TP, FP, FN, and TN in patients with PICs, categorized by study. For each study, pooled sensitivity and specificity were derived, along with the associated confidence interval (CI). To synthesize diagnostic precision across multiple studies, we computed the hierarchical summary receiver operating characteristic (HSROC) curve. The construction of HSROC curves involves initial fitting of a hierarchical model to the collected data from all tests or studies, considering the variance in test accuracy among different studies and the correlation between sensitivity and specificity within each study. Furthermore, to evaluate publication bias, funnel plots were assembled and Deeks’ test was used. Fagan’s nomogram was also used to determine the post-test likelihood of a disease, given a positive or negative test outcome. To investigate the factors contributing to the heterogeneity in the sensitivity and specificity of presepsin, subgroup analyses were performed. These analyses were stratified based on several criteria: the type of surgery (i.e., abdominal surgery); the presepsin cut-off value (i.e., categorized as either less than 400 pg/ml or greater than 400 pg/ml); and the timing of measurement (i.e., measurement of presepsin levels during postoperative days 1 to 3). Statistical analyses were conducted using STATA (version 15.1), incorporating the MIDAS module, and Review Manager (version 5.3). A two-sided *p*-value <0.05 was considered to indicate statistical significance.

## 3 Results

### 3.1 Study selection and characteristics of studies

**Figure 1:**
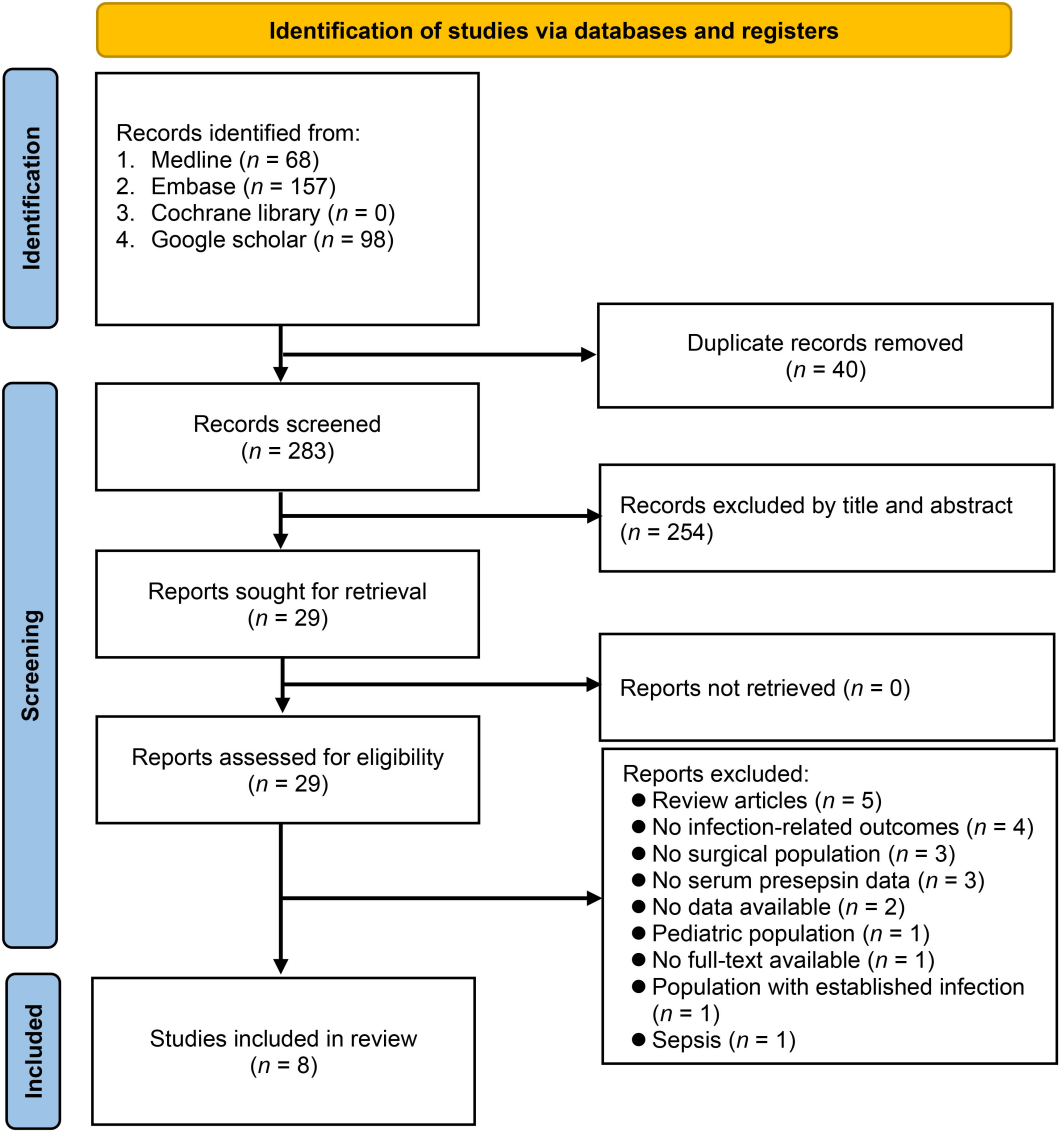
Flow chart of study selection.

**Figure 1** illustrates the study selection process. A total of 323 potential studies were initially retrieved from the four databases. After the removal of 40 duplicate entries, 283 studies were screened by title and abstract. Then, after excluding 254 studies that did not meet the inclusion criteria, the remaining 29 studies were thoroughly reassessed via full-text screening. Additional 20 studies were excluded due to various reasons (e.g., lack of specific data), resulting in the inclusion of eight studies (1, 15, 16, 19, 21–24) in this meta-analysis. The inter-rater reliability for the assessment of study inclusion was 0.84, indicating a very good agreement between the two investigators.

This meta-analysis included eight studies published between 2015 and 2022 that evaluated presepsin as a biomarker for PICs (**Table 1**). The studies were conducted in several countries, including Japan (n = 6) (1, 15, 21–24), Russia (n = 1) (19), and China (n = 1) (16). The total number of patients across all studies was 984, with sample sizes ranging from 13 to 490 patients. The patients underwent various types of surgery, including colorectal, cardiac, hepatobiliary–pancreatic, and spinal surgeries, gastrectomy, esophagectomy, and liver transplantation. The mean or median age of the patients in eight studies was reported to range from 44 to 71.5 years; however, one study did not provide detail regarding age (16). The percentage of male patients varied from 30.8% to 80% across eight studies, with one study lacking relevant data (19). Furthermore, the infection rates after surgery widely varied between studies from 6.9% to 38.5%. Presepsin was measured at various postoperative time points from days 1 to 7. The cutoff values used for presepsin to diagnose PCIs ranged from 258 to 1,375 pg/mL. The reported sensitivity of presepsin for diagnosing infections ranged from 60% to 100%, whereas specificity ranged from 64.2% to 91.4%.

**Table 1:** Characteristics of studies (n=9).

Studies	Age (years)	Male (%)	*n*	Infection rate (%)	Surgery	Cut-off value of presepsin (pg/ml)	Time of measurement	Sensitivity (%)	Specificity (%)	Country
Amanai 2022	63.7 vs. 69.1	69.3	114	23.7	Colorectal surgery	294	POD 6	70	81.8	Japan
Imai 2022	71.5	69.4	108	16.7	Gastrectomy	298	POD 3	83.3	83.3	Japan
Popov 2015	58	na	51	37.3	Cardiac surgery	702	POD 1	72	66	Russian Federation
Suzuki 2021	69.2	58.9	73	27.4	Cardiac surgery	347	POD 1	75	64.2	Japan
Takeuchi 2020	72	80	30	33.3	Esophagectomy	668	POD 7	60	85	Japan
Yao 2020	70	56.2	105	14.3	HBP surgery	620	POD 3	93.3	89.2	Japan
Yokose 2021	44	30.8	13	38.5	Liver transplantation	1375	POD 7	100	85.7	Japan
Zhu 2022	>18	55.1	490	6.9	Spinal surgery	258	POD 1	71.9	91.4	China

HBP, hepato-biliary-pancreatic; POD, postoperative day; na, not available.

### 3.2 Risk of bias

**Figure 2:**
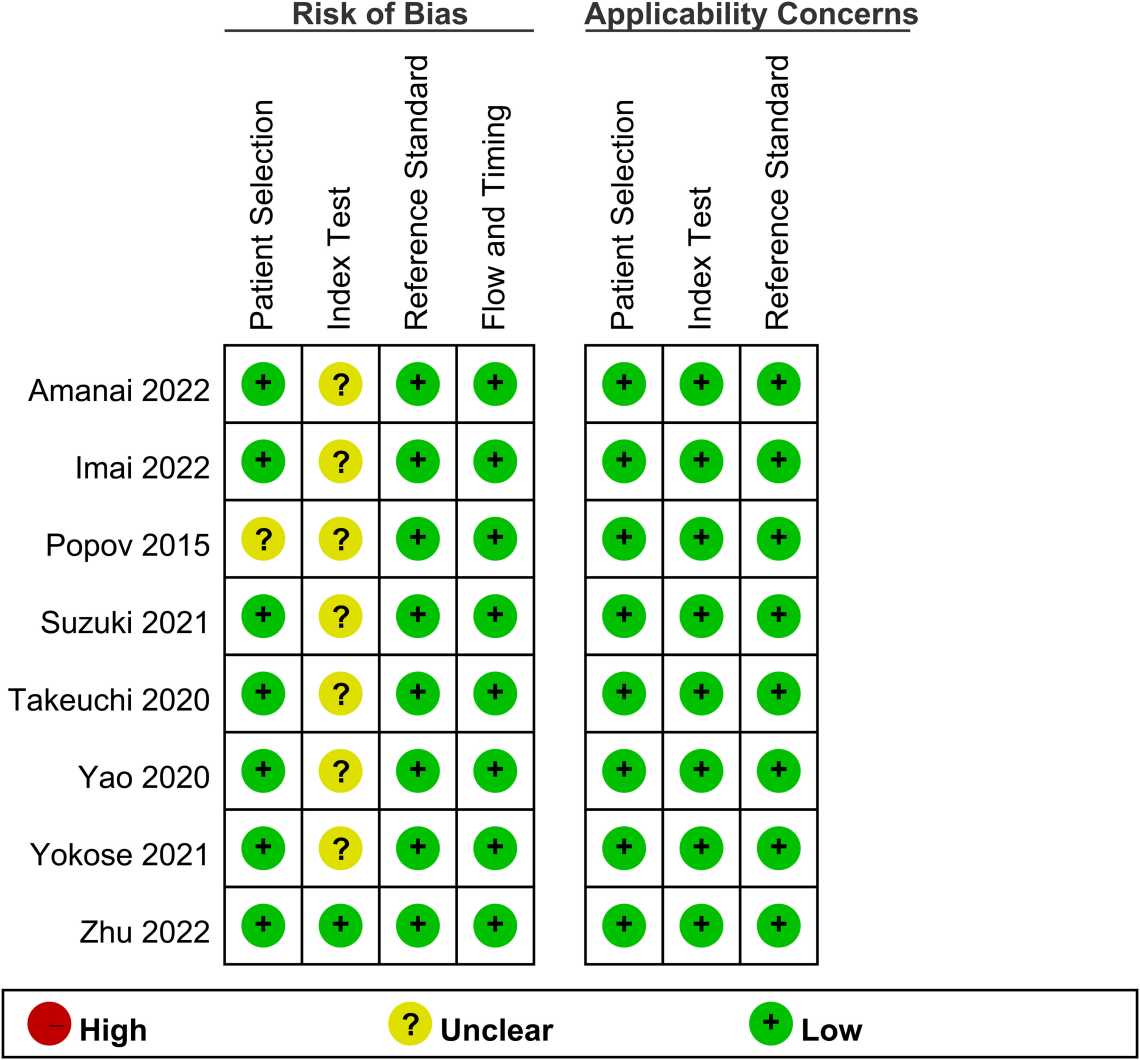
Summary of risk of bias for the included studies.

All studies were found to possess a low risk of bias in terms of the reference standard, flow and timing, and applicability (**Figure 2**) (1, 15, 16, 19, 21–24). However, as regards the index test domain, the risk of bias in all studies was unclear due to the lack of predefined cutoff values. Apart from the index test domain, one study (19) had an unclear risk of bias in terms of patient selection as it did not provide comprehensive inclusion/exclusion criteria.

### 3.3 Efficacy of presepsin, procalcitonin, and CRP in predicting postsurgical infection

**Figure 3:**
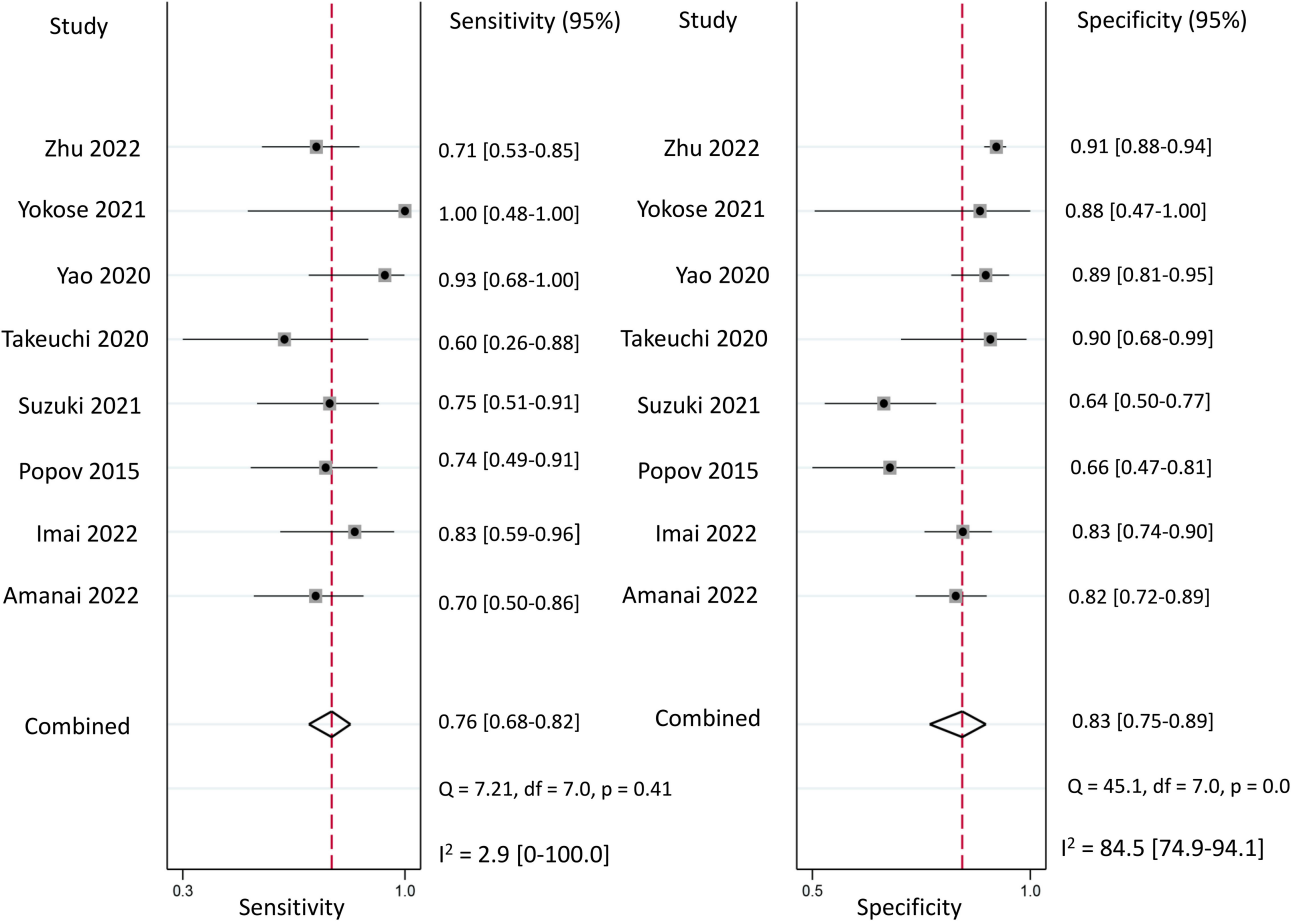
Forest plot showing the pooled sensitivity and specificity of presepsin for the diagnosis of postoperative infectious complications (PICs).

**Figure 4:**
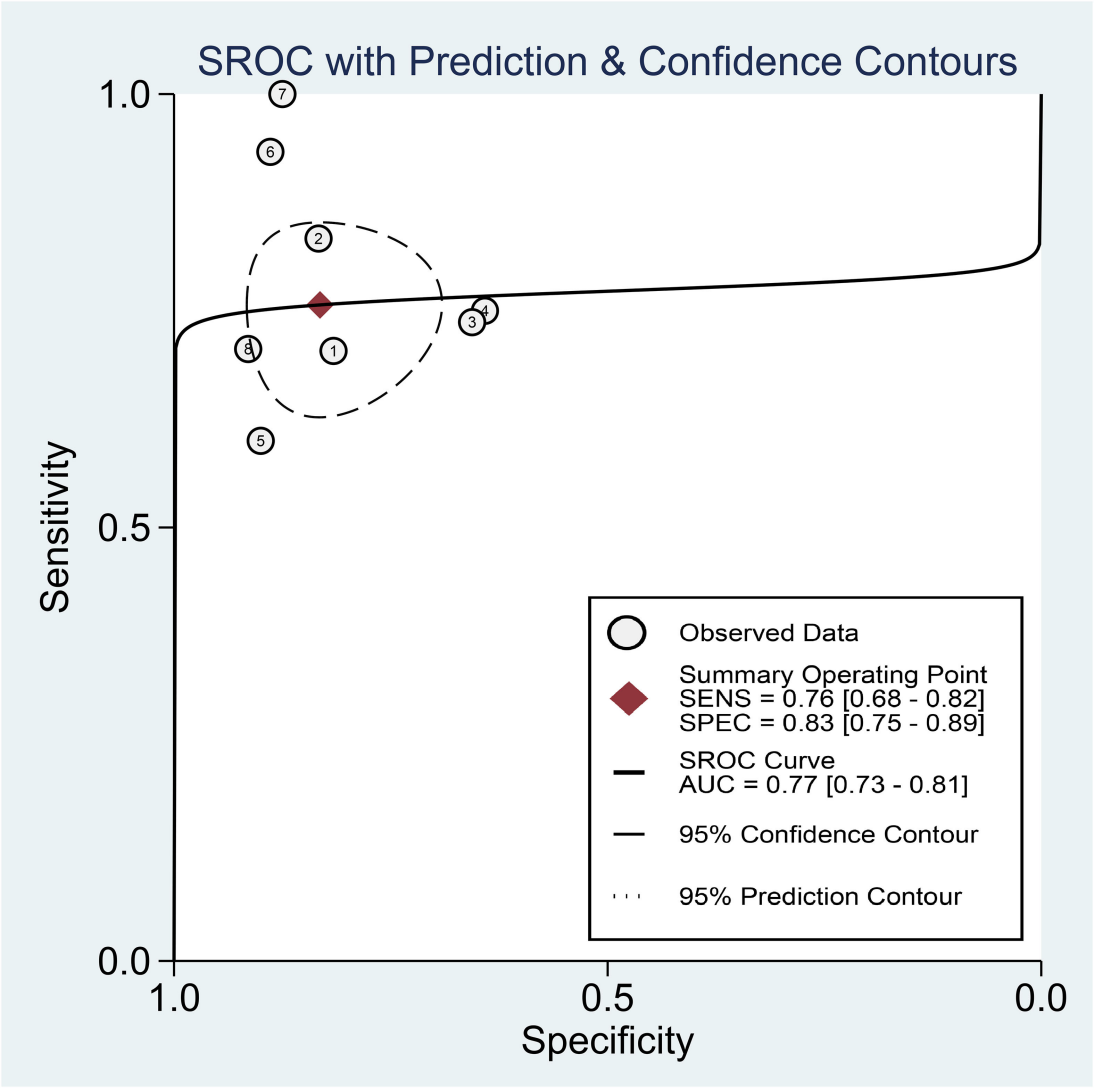
Hierarchical summary receiver operating characteristic curve of presepsin for the diagnosis of postoperative infectious complications (PICs).

**Figure 5:**
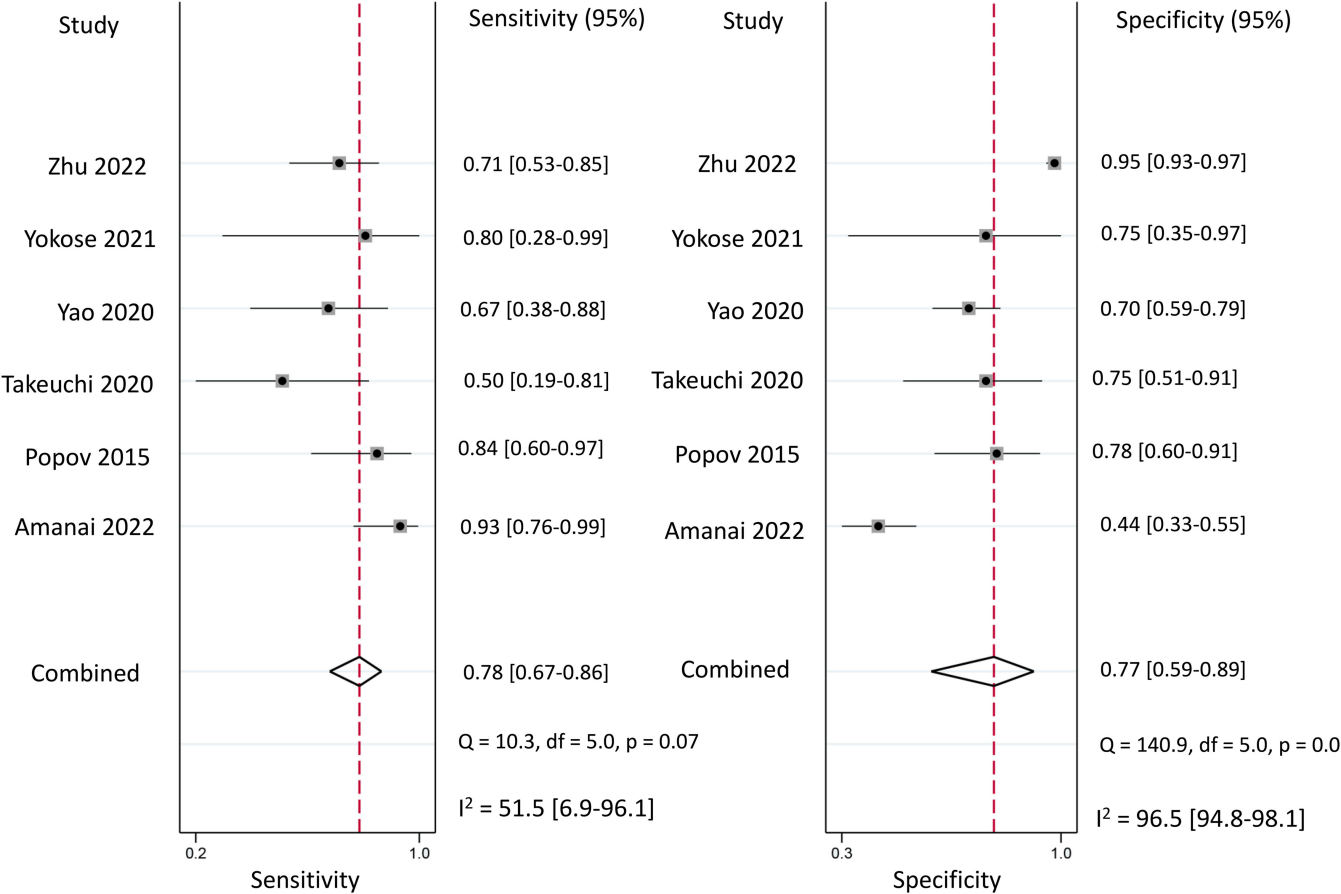
Forest plot showing the pooled sensitivity and specificity of procalcitonin for the diagnosis of postoperative infectious complications (PICs).

**Figure 6:**
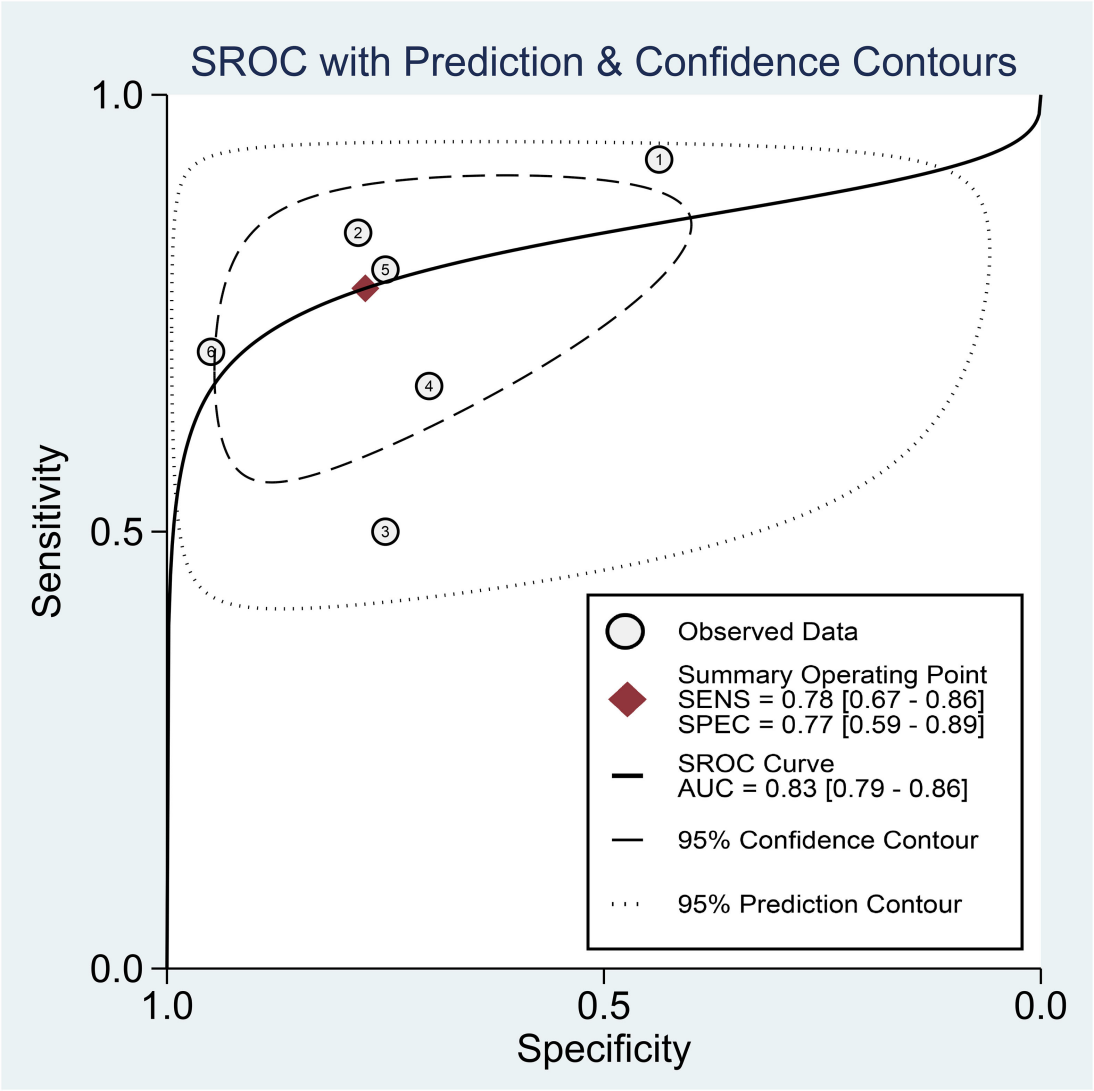
Hierarchical summary receiver operating characteristic curve of procalcitonin for the diagnosis of postoperative infectious complications (PICs).

**Figure 7:**
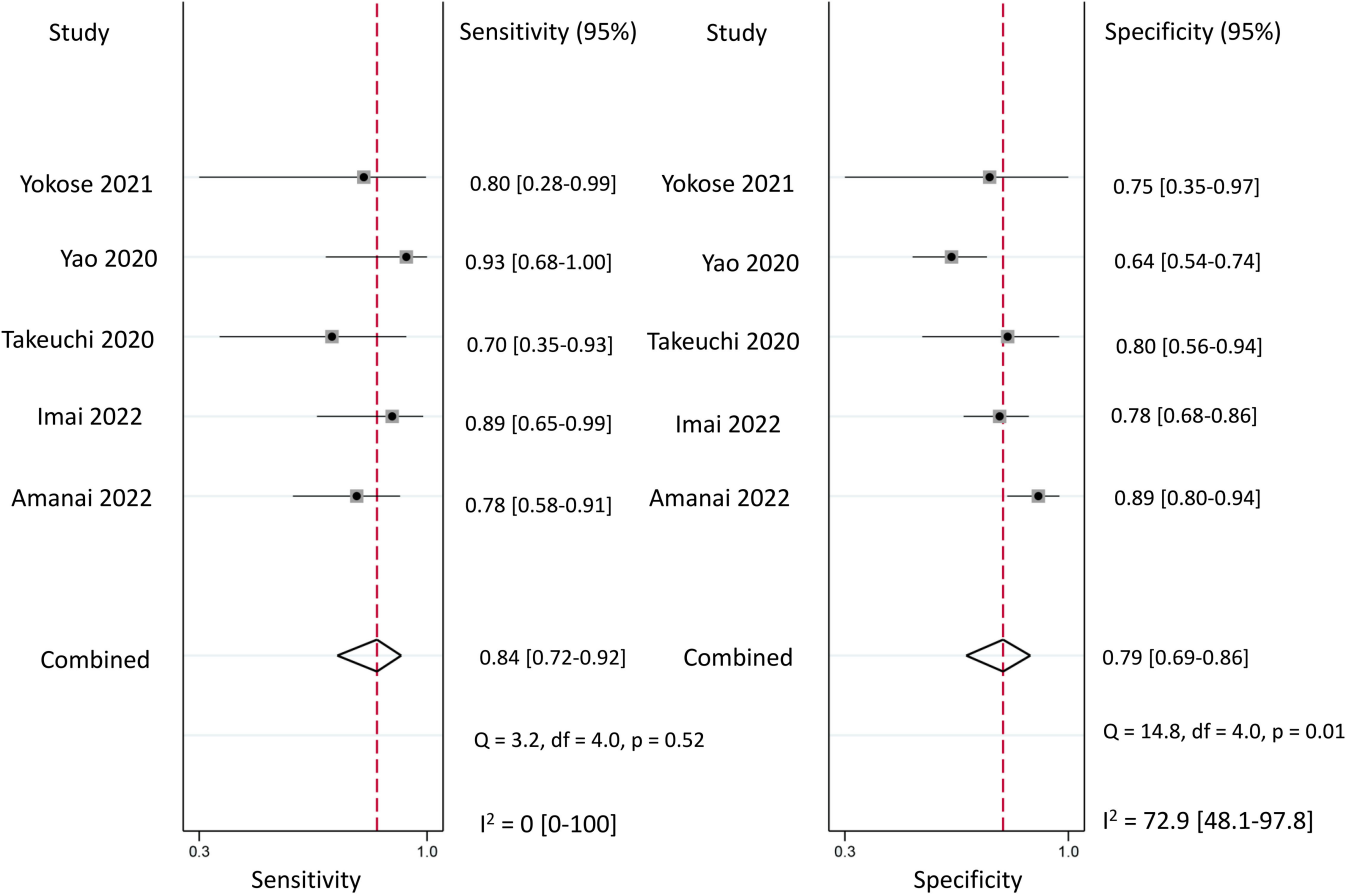
Forest plot showing the pooled sensitivity and specificity of C-reactive protein (CRP) for the diagnosis of postoperative infectious complications (PICs).

**Figure 8:**
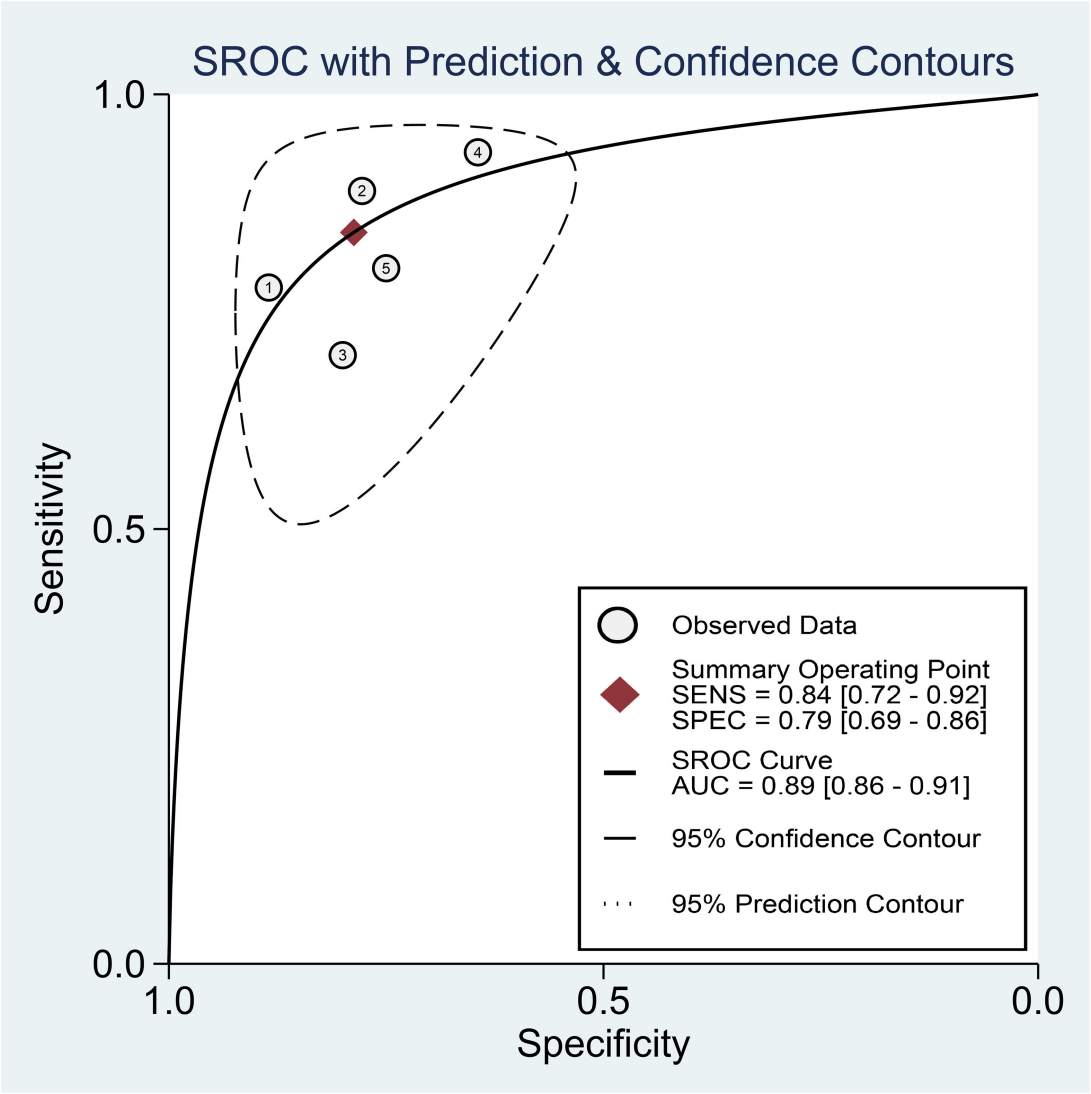
Hierarchical summary receiver operating characteristic curve of C-reactive protein (CRP) for the diagnosis of postoperative infectious complications (PICs).

Regarding the diagnostic efficacy of presepsin for PIC, extraction of data from eight studies enabled the computation of pooled sensitivity of 76% (95% CI 68%–82%, I^2^ = 2.9%) and specificity of 83% (95% CI 75%–89%, I^2^ = 84.5%) (**Figure 3**) (1, 15, 16, 19, 21–24). The HSROC curve showed an area under the curve (AUC) of 0.77 (95% CI 0.73–0.81) (**Figure 4**). As for procalcitonin, a synthesis of six studies yielded a combined sensitivity and specificity of 78% (95% CI 67%–86%) and 77% (95% CI 59%–89%), respectively (**Figure 5**) (1, 16, 19, 22–24). The corresponding AUC, derived from the HSROC curve, was 0.83 (95% CI 0.79–0.86) (**Figure 6**). As for CRP, analysis of five studies revealed pooled sensitivity and specificity of 84% (95% CI 72%–92%) and 79% (95% CI 69%–86%), respectively (**Figure 7**) (1, 15, 22–24). The associated AUC, based on the HSROC curve, stood at 0.89 (95% CI 0.86–0.91) (**Figure 8**).

### 3.4 Risk of publication bias

**Figure 9:**
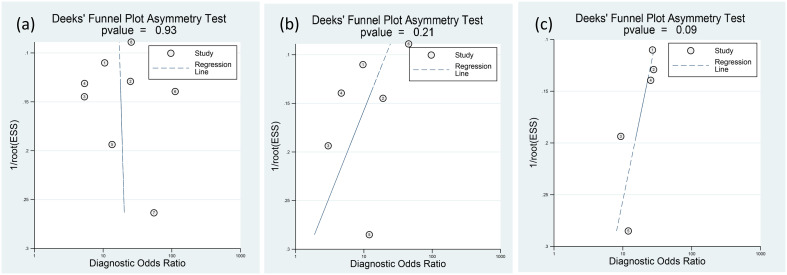
Deeks’ funnel-plot asymmetry test for **(A)** presepsin, **(B)** procalcitonin, and **(C)** C-reactive protein (CRP). The three biomarkers have low risks of publication bias.

The funnel plots depicting presepsin, procalcitonin, and CRP are presented in **Figure 9**. Analysis of these plots revealed a low risk of publication bias for each of the three biomarkers, with *p*-values of 0.93, 0.21, and 0.09 for presepsin, procalcitonin, and CRP, respectively.

### 3.5 Fagan plots

**Figure 10:**
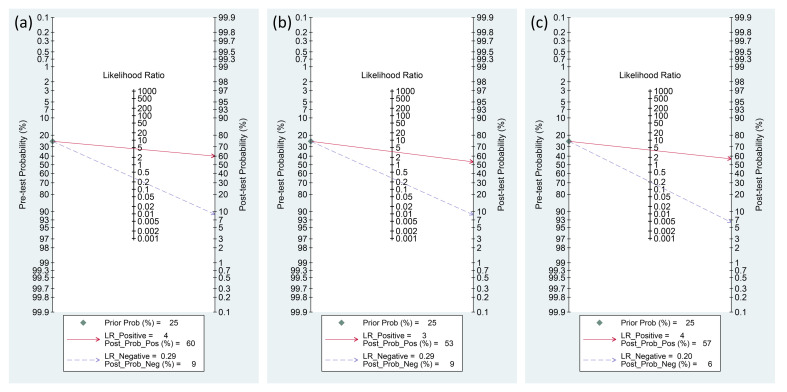
Fagan diagrams for the diagnosis of PICs: **(A)** presepsin, **(B)** procalcitonin, and **(C)** C-reactive protein (CRP).

The Fagan graph was plotted to show the relationship among the pretest probabilities, likelihood ratio, and post-test probabilities for three biomarkers (**Figure 10**). A positive likelihood ratio of four indicates that an individual with an infection is four times more likely to have a positive test result than an individual without an infection. Given a pretest probability of 25%, the post-test probability of receiving a positive test result for presepsin is 60%. Conversely, a negative likelihood ratio of 0.29 decreases the post-test probability to 9% for a negative test result. The Fagan plots show that the positive and negative likelihood ratios were similar across the three biomarkers. In other words, the post-test probabilities for positive and negative test results were comparable among the three biomarkers.

### 3.6 Subgroup analysis

To examine the heterogeneity in the sensitivity and specificity of presepsin, subgroup analyses were conducted. In studies focusing on abdominal surgery, the combined sensitivity and specificity were 80% (95% CI: 65–90%, I^2^ = 40.94%) and 85% (95% CI: 80–89%, I^2^ = 0%), respectively (**Supplemental Figure 1**). Conversely, in other subgroup analyses based on the presepsin cut-off value or the timing of measurement (i.e., postoperative days 1 to 3) (**Supplemental Figures 2**–**4**), the heterogeneity for specificity remained significant, with an I^2^ range of 69.88-91.49%. These findings suggest that the type of surgery might be the primary source of variability in specificity when using presepsin to predict PICs.

## 4 Discussion

This meta-analysis aimed to systematically evaluate and compare the diagnostic efficacies of serum presepsin, procalcitonin, and CRP for PICs. The identification of optimal biomarker can guide decision-making regarding further diagnostic workup for suspected PIC. Furthermore, this meta-analysis of eight studies revealed that presepsin had pooled sensitivity and specificity of 76% and 83%, respectively, with an AUC of 0.77 from the HSROC curve. Meanwhile, an analysis of six studies on procalcitonin revealed a combined sensitivity of 78% and specificity of 77%, with an HSROC-derived AUC of 0.83. For CRP, data from five studies yielded pooled sensitivity of 84% and specificity of 79%, with the HSROC curve indicating an AUC of 0.89. The present meta-analysis represents the initial extensive evaluation of presepsin’s diagnostic efficacy in detecting PICs, thereby offering invaluable information to healthcare providers regarding the employment of this biomarker in clinical settings.

Diagnosing PICs remains a complex issue in surgical patient care. Identifying PICs can be difficult as both tissue damage and infection can manifest similar clinical and laboratory signs of inflammation. The situation is more complicated for cardiac surgery patients, as systemic inflammatory response syndrome related to cardiopulmonary bypass can show signs of PICs (25). Furthermore, routine systemic antibiotics, particularly in the intensive care unit, can adversely affect the efficacy of blood cultures (26). As the diagnostic criteria for PICs (e.g., hospital-acquired pneumonia) are sometimes nonspecific, a previous study explored the reduction of infection rates through extended antibiotic prophylaxis for high-risk cardiac surgery patients (27). The findings indicated that prolonged postoperative prophylaxis with antibiotics did not reduce infection complication rates (27). As such, extending antibiotic prophylaxis might be unnecessary and can sometimes lead to an increase in the number of antibiotic-resistant pathogens (28). Conversely, promptly diagnosing and addressing PICs is crucial to achieve optimal patient outcomes.

Presepsin, also known as soluble CD14 subtype, is a fragment derived from the CD14 receptor (29–31). This 13-kDa molecule functions as a soluble pattern recognition receptor and is released into the blood during infection and inflammation (29–31). The levels of presepsin can be affected by factors such as acute pancreatitis, age (particularly in neonates and older individuals), and burns (32–34). In addition, given that presepsin is processed by the glomerulus of the kidney and reabsorbed in the proximal tubules, any condition impacting renal filtration influences plasma presepsin concentrations. In the current meta-analysis, studies that included patients exhibiting evident symptoms of infection before surgery or those with end-stage renal disease were omitted, given that these conditions influence presepsin levels (35, 36). While previous studies reported elevated presepsin levels in patients with viral meningitis and fungemia (37, 38), the diagnostic accuracy for sepsis related to parasites, viruses, and fungi remains to be firmly established. During the onset of sepsis, the levels of presepsin elevate early (39, 40), making it a valuable indicator for detecting and predicting the course of sepsis (13). While surgical trauma has not been associated with elevated presepsin levels (1, 15, 22), the utility of presepsin in the diagnosis of PICs remain unclear due to the lack of consolidated evidence.

When diagnosing PICs, our meta-analysis revealed that presepsin has combined sensitivity of 76% and specificity of 83%. The AUC from the HSROC curve was 0.77, indicating its potential diagnostic accuracy. A recent meta-analysis of 33 studies revealed that presepsin, in the diagnosis of sepsis, exhibited pooled sensitivity of 0.86, pooled specificity of 0.79, and an AUC of 0.90 (13). It seems that presepsin can be considered as a useful biomarker for both PICs and sepsis. In our meta-analysis, the reported cutoff values for presepsin ranged between 258 and 1,375 pg/mL. These findings are consistent with the results of a recent meta-analysis centered on the diagnostic capability of presepsin for sepsis, wherein the cutoff values spanned from 89.26 to 1,315 pg/mL. The extensive variation in these cutoff values could be attributed to disparities in disease severity, study methodologies, and clinical settings (13). To determine a clinically relevant cutoff value, further research targeting specific patient subgroups and the efficacy of presepsin is essential. To the best of our knowledge, our meta-analysis is the first to explore the utility of presepsin in the diagnosis of PICs, thereby bridging the knowledge gap concerning the clinical application of presepsin.

Our analysis on the diagnostic efficacy of procalcitonin for PICs revealed a combined sensitivity of 78% and specificity of 77%, with an HSROC-derived AUC of 0.83. Existing research has delved into the diagnostic efficacy of procalcitonin in PICs. A previous meta-analysis of ten studies on major gastrointestinal surgery revealed the diagnostic accuracy of procalcitonin, presenting pooled sensitivity of 72%, specificity of 62%, and AUC of 0.766 (41). Another meta-analysis focusing on cardiac surgery revealed pooled sensitivity and specificity of 81% and 78%, respectively, with the AUC being 0.87 (42). In addition, a meta-analysis centered on pancreatic surgery showed that on postoperative day 3, the pooled sensitivity, specificity, and AUC for procalcitonin were 74%, 79%, and 0.8453, respectively (43). However, when the diagnostic efficacy for postoperative orthopedic infections was evaluated, the results were less promising, with pooled sensitivity and specificity of 67.3% and 69.4%, respectively (44). The sensitivity, specificity, and AUC for procalcitonin in the current meta-analysis may be comparable with those in the previous meta-analyses (41–43).

CRP is commonly used as an indicator of inflammation owing to its accuracy, versatility, and cost-effectiveness. For CRP, our analysis of five studies yielded pooled sensitivity of 84% and specificity of 79%, with the HSROC curve indicating an AUC of 0.89. Several meta-analyses have examined the diagnostic efficacy of CRP for PICs across different surgical procedures. An analysis of 11 studies on elective colorectal surgery revealed pooled sensitivity of 75% and specificity of 72% for CRP, with an AUC of 0.8 (45). Another meta-analysis centered on major abdominal surgery highlighted pooled sensitivity and specificity of 77% each for CRP on postoperative day 3, along with an AUC of 0.87 (46). Meanwhile, a meta-analysis on bariatric surgery reported pooled sensitivity of 81% and specificity of 91% for CRP on postoperative day 3, with an AUC of 0.81 (47). Although our meta-analysis included a variety of surgical procedures, our findings on the diagnostic efficacy of CRP were similar to those of previous meta-analyses (45–47). CRP levels can increase under various inflammatory conditions, not only due to infections but also due to invasive incidents like surgeries and trauma (48). As noted in a previous study, postoperative CRP levels increased in both infected and noninfected patients, complicating the interpretation of CRP values (1). As a result, any elevation in CRP levels suggesting infection should be approached with caution in clinical practice.

HSROC-derived AUC indicates that CRP may offer the most favorable diagnostic efficacy for PICs, followed by procalcitonin and presepsin. The inferiority of presepsin in terms of efficacy can be attributed to the lack of accuracy when acute kidney injury develops (49). Most studies in our meta-analysis focused on major surgeries that might induce postoperative renal dysfunction (50–52), potentially confounding diagnostic efficacy. Nevertheless, presepsin exhibited higher specificity than other biomarkers, likely due to its tendency of not changing immediately post-surgery but of increasing with infectious complications (1). Based on these findings, combining different biomarkers might enhance diagnostic efficacy, but this approach needs to be studied further.

The present meta-analysis has several limitations that need to be considered. First, the studies are geographically concentrated in Asian regions, particularly Japan and China, potentially affecting the universality of the findings. Second, the total sample size of 984 patients, while inclusive of all available evidence, is relatively small, raising concerns about generalizability. Third, the diversity of surgical procedures, ranging from colorectal to cardiac, introduces heterogeneity in postoperative infection risk profiles, indicating the benefit of specialized subgroup analyses. Fourth, the timing of presepsin level measurements in the postoperative period, ranging from days 1 to 7, might influence diagnostic accuracy. Furthermore, the significant discrepancies in the reported cutoff values across studies significantly impede the practical use of presepsin in clinical practice. Finally, this meta-analysis focused solely on diagnostic accuracy, and did not evaluate the potential effects of presepsin testing on clinical management and patient outcomes such as morbidity and mortality. Further research is needed to determine the practical implications and clinical utility of presepsin as a diagnostic tool for PICs.

## 5 Conclusion

The present meta-analysis of eight studies on PICs revealed that presepsin had pooled sensitivity of 76%, specificity of 83%, and AUC of 0.77. CRP exhibited the highest diagnostic efficacy for PICs, followed by procalcitonin and presepsin. Nonetheless, presepsin exhibited greater specificity than the other biomarkers. Combined use of these biomarkers as supplementary tests may lead to the early identification of infection. However, considering the scarcity of studies and the fact that the majority were carried out in Asian countries, it is crucial to conduct additional research, particularly in non-Asian regions, to validate these findings and to establish optimal cutoff values for presepsin’s utility.

## Data availability statement

The original contributions presented in the study are included in the article/**Supplementary Material**. Further inquiries can be directed to the corresponding author.

## Author contributions

C-YL: Methodology, Writing – original draft, Writing – review & editing. C-LK: Formal analysis, Writing – original draft, Writing – review & editing. K-CH: Conceptualization, Writing – original draft, Writing – review & editing. J-YW: Formal analysis, Methodology, Writing – original draft, Writing – review & editing. H-CH: Software, Validation, Writing – original draft, Writing – review & editing. C-HY: Data curation, Validation, Writing – original draft, Writing – review & editing. W-TC: Conceptualization, Resources, Writing – original draft, Writing – review & editing. P-HF: Formal analysis, Methodology, Writing – original draft, Writing – review & editing. I-WC: Conceptualization, Supervision, Visualization, Writing – original draft, Writing – review & editing.
