# Supplementary material for: Diagnostic efficacy of serum presepsin for postoperative infectious complications: a meta-analysis
Source: Front Immunol. 2023 Dec 12;14:1320683. doi: 10.3389/fimmu.2023.1320683 (PMC10750271; doi:10.3389/fimmu.2023.1320683)

**Supplemental Table 1.** Search strategy for MEDLINE

| 1 | ("Postoperative" or "Surgery" or "Surgical Procedure" or "Operative Procedure" or "General anesthesia" or "Operation" or "Surgical").mp. |
| --- | --- |
| 2 | exp "Surgical Procedures, Operative"/ |
| 3 | ("Presepsin" or "sCD14-ST" or "Soluble CD14 subtype").mp. |
| 4 | ("Infection" or "Sepsis" or "Abscess" or "Cellulitis" or "Pneumonia" or "Osteomyelitis" or "Cystitis" or "Urethritis" or "Pyelonephritis" or "Surgical site infection" or "Septicemia" or "nosocomial infection" or "Hospital Infection" or "peritonitis").mp. |
| 5 | exp "Infections"/ or exp "Sepsis"/ or exp "Surgical Wound Infection"/ or exp "Cross Infection"/ or exp "Urinary Tract Infections"/ or exp "Pneumonia"/ |
| 6 | (1 or 2) and 3 and (4 or 5) |
| 7 | ROC curve.mp. |
| 8 | 6 and 7 |

**Supplemental Figure 1.** Subgroup analysis based on abdominal surgery


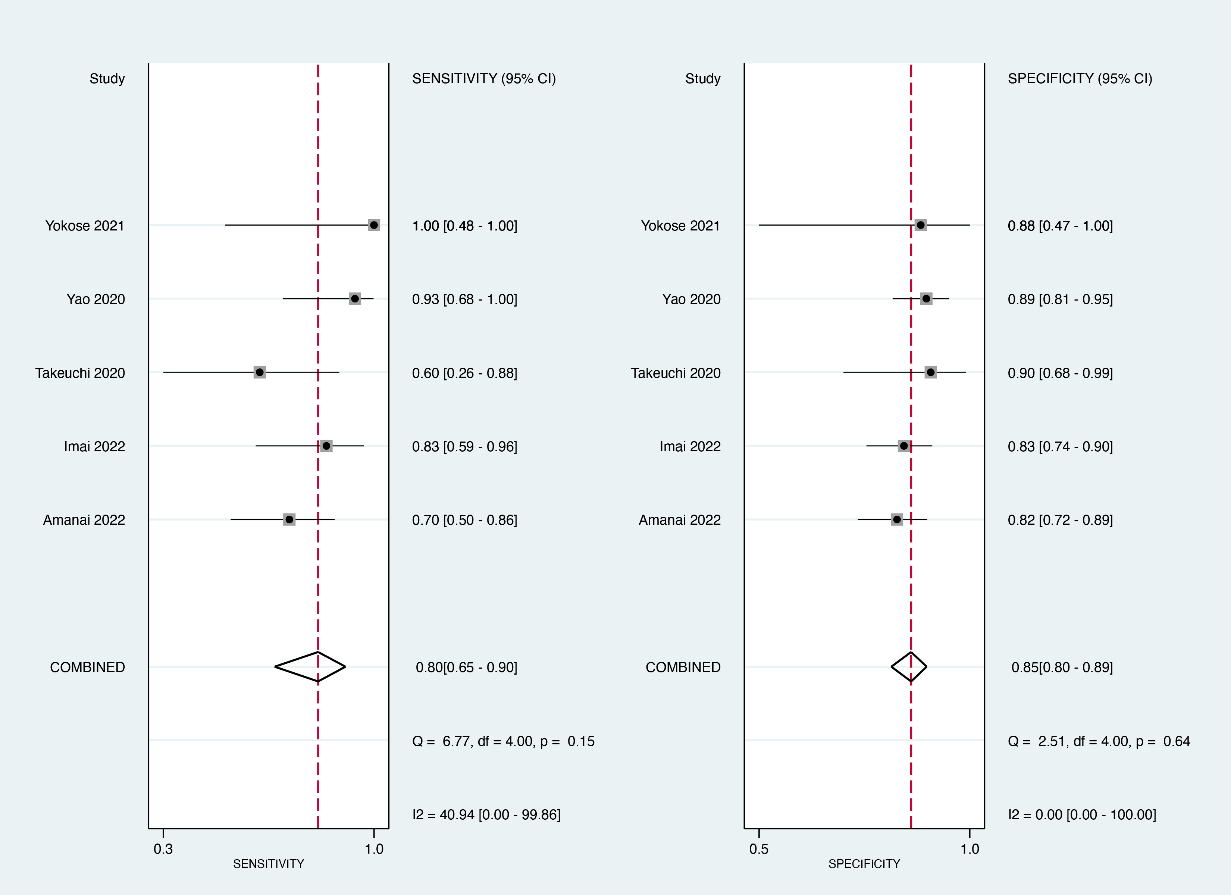


**Supplemental Figure 2.** Analyzing Subgroups with Presepsin Levels < 400 pg/ml
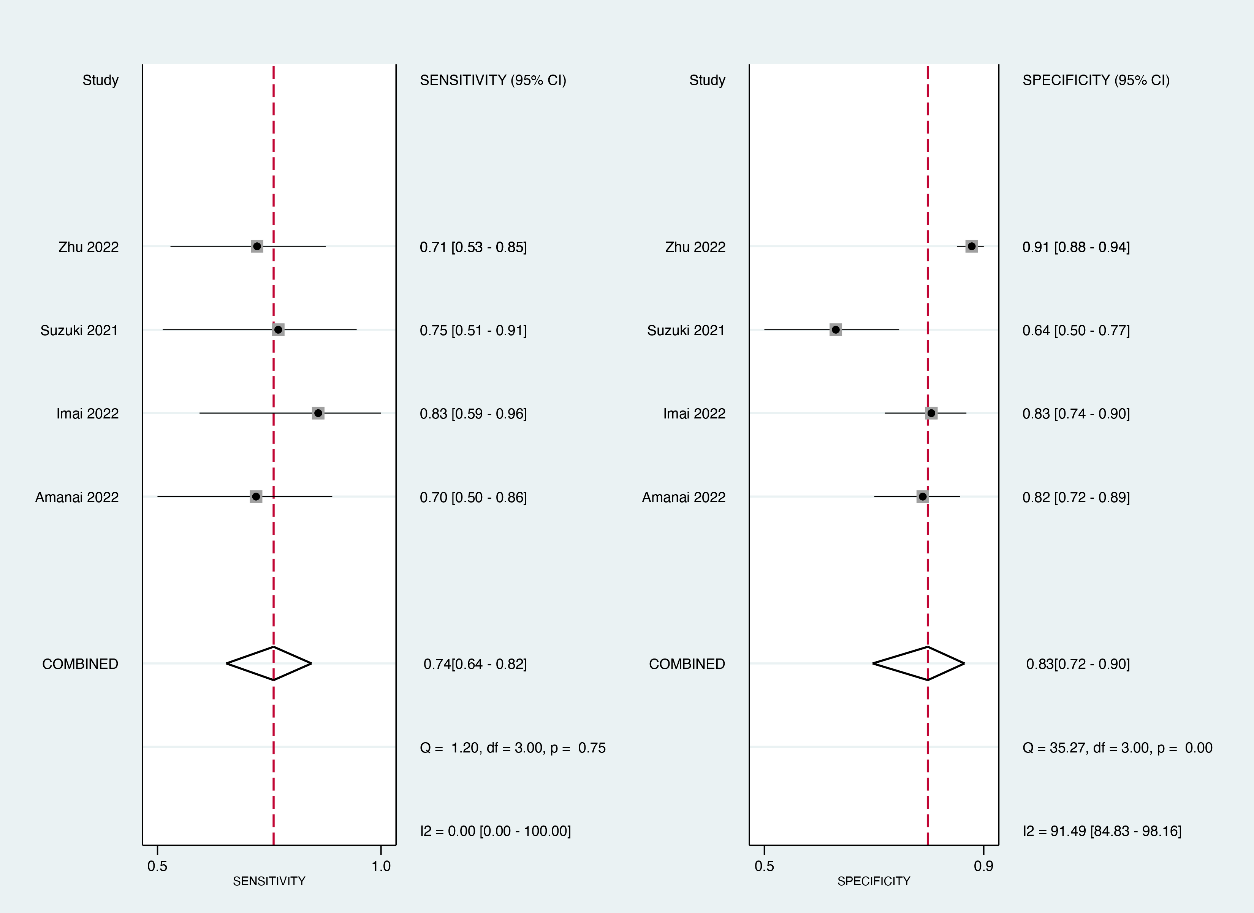


**Supplemental Figure 3.** Analyzing Subgroups with Presepsin Levels > 400 pg/ml


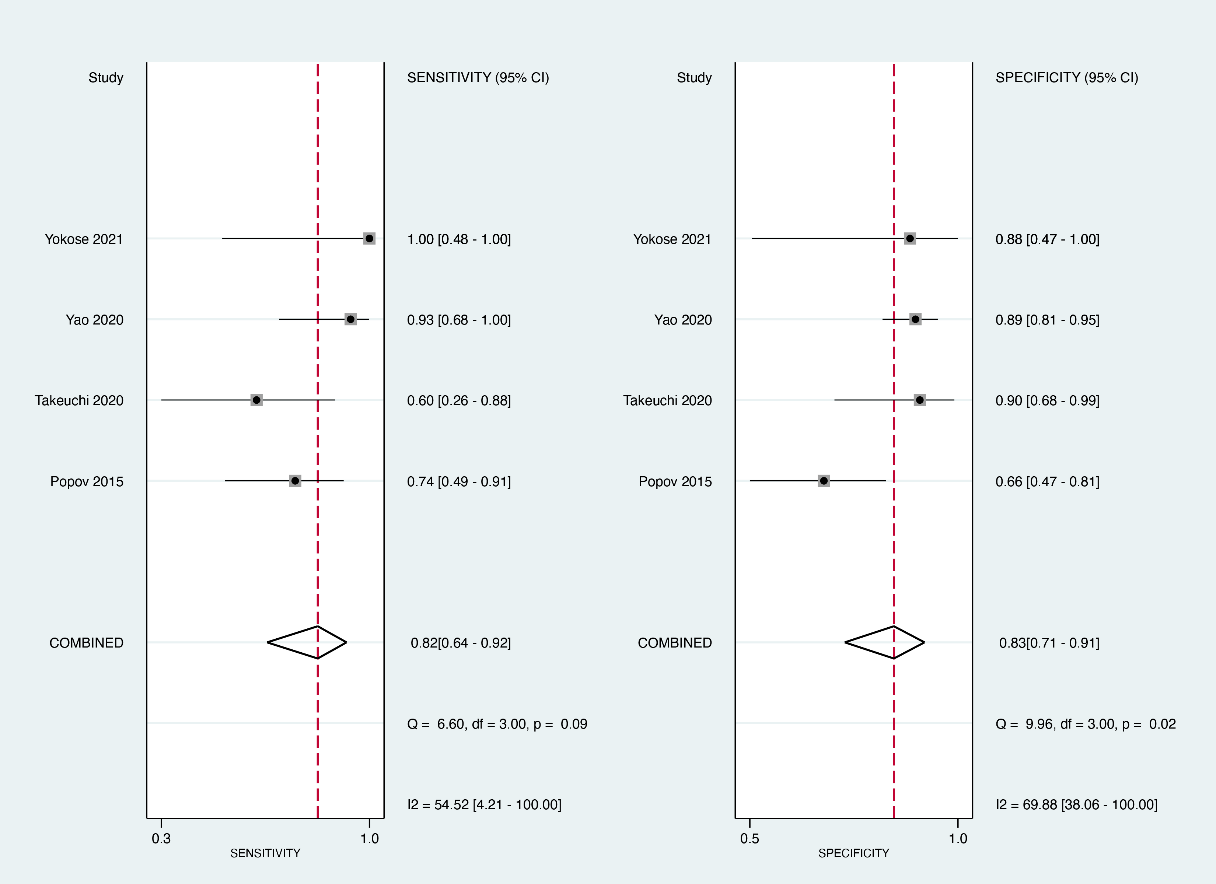


**Supplemental Figure 3.** Subgroup analysis based on time of measurement (postoperative days 1 to 3)


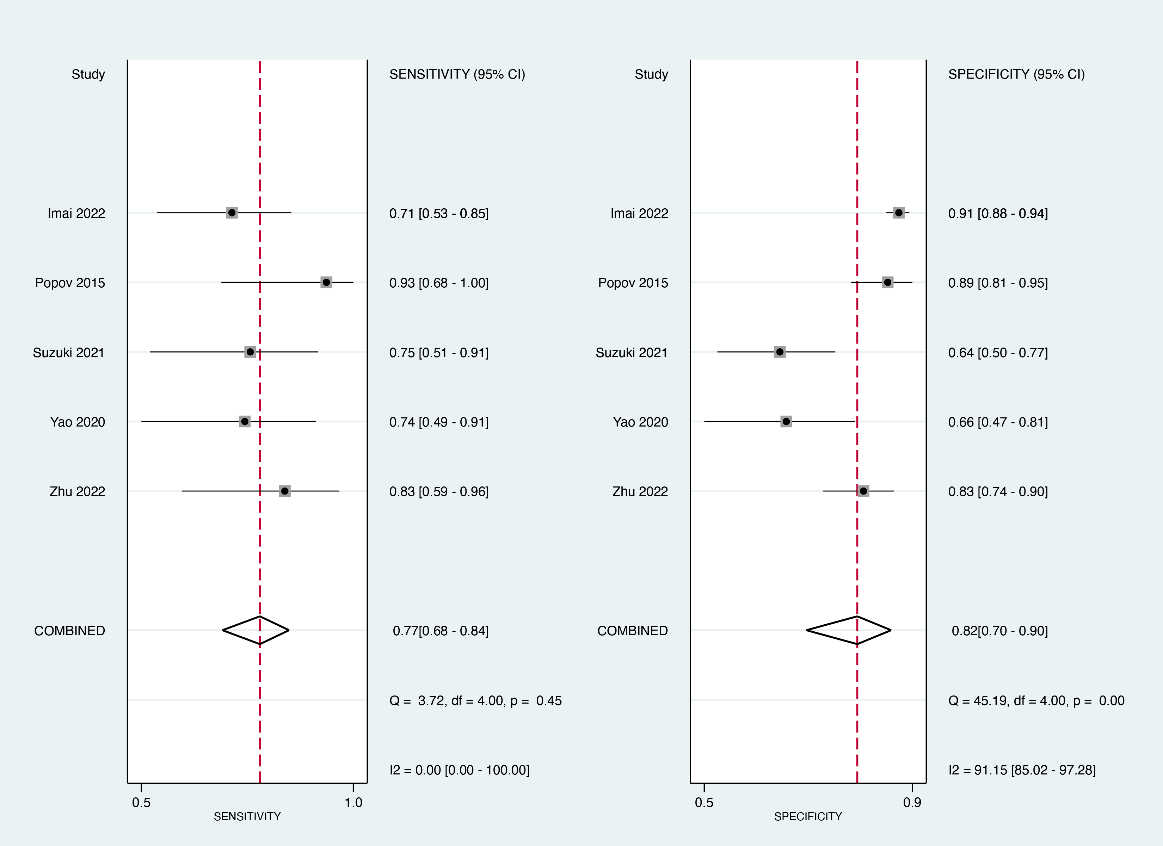

Supplement: Supplementary file 1 [file DataSheet_1.docx]
